# Bernard Soulier Syndrome Misdiagnosed and Treated as Immune Thrombocytopenia Purpura: A Case Report

**DOI:** 10.7759/cureus.103614

**Published:** 2026-02-14

**Authors:** Misha Jannat, Abdul Raffeh Basit, Saad Khurram, Rayshum Jarral, Muhammad Hassan Qureshi

**Affiliations:** 1 Medicine and Surgery, Queen Mary University of London, London, GBR; 2 Medicine, CMH (Combined Military Hospital) Lahore Medical College and Institute of Dentistry, Lahore, PAK; 3 Internal Medicine, CMH (Combined Military Hospital) Lahore Medical College and Institute of Dentistry, Lahore, PAK; 4 Family Medicine, Cambridgeshire and Peterborough NHS Foundation Trust, Peterborough, GBR; 5 Family Medicine, Shalamar Medical and Dental College, Lahore, PAK; 6 Public Health and Policy, Anglia Ruskin University, Cambridge, GBR

**Keywords:** bernard soulier syndrome, immune thrombocytopenia purpura, itp management, misdiagnosed case, total splenectomy

## Abstract

Bernard Soulier syndrome (BSS) is a rare autosomal recessive disorder that presents with giant platelets, prolonged bleeding time, and mucocutaneous bleeding. As it is a rare disorder and shares overlapping clinical features with other bleeding disorders, it is often misdiagnosed as immune thrombocytopenia purpura (ITP), which can result in inappropriate and aggressive management. This report explores the case of a young female patient who presented with recurrent epistaxis and ecchymotic episodes since childhood. She was misdiagnosed and treated for ITP, and due to refractory symptoms, she underwent splenectomy and experienced temporary symptom improvement. However, the symptoms returned, and further workup with ristocetin and flow cytometry confirmed her diagnosis of BSS. This case underscores the importance of considering an alternative diagnosis for ITP, particularly when the presentation is atypical and the treatment response is also unusual. Additionally, it examines whether splenectomy is associated with better outcomes for these patients.

## Introduction

Bernard Soulier syndrome (BSS) is a rare autosomal recessive qualitative platelet disorder. It affects less than one person per million people, but this may be higher as BSS is often misdiagnosed [1]. It occurs due to mutations in the *GP1BA*, *GP1BB*, or *GP9* genes. This results in defective expression of the GPIb/IX/V complex on the platelet surface, resulting in decreased interaction between GPIbα with von Willebrand factor (vWF), which leads to failure of primary hemostasis [2-4]. It presents as mucocutaneous bleeding, epistaxis, bruising, and gum bleeding, often starting in early childhood [5]. 

BSS is characterized by thrombocytopenia and giant platelets. In this disorder, platelets do not agglutinate in the presence of ristocetin, and the diagnosis is made by flow cytometry [6]. The differential diagnosis for BSS is May-Hegglin anomaly, Glanzmann Thrombasthenia, von Willebrand disease, or immune thrombocytopenia purpura (ITP) [6]. As BSS is rare and presents similarly to other platelet disorders, it is often misdiagnosed, which can lead to the use of treatment modalities like splenectomy (for ITP) that are unnecessary and non-curative [7]. 

This case report highlights the diagnostic challenges that physicians face in diagnosing and treating rare inherited bleeding disorders and how they could be misdiagnosed and mistreated initially. 

## Case presentation

A 17-year-old female patient, with a long-standing history of mucocutaneous bleeding since childhood, presented with hepatitis C.

The patient has had recurrent episodes of epistaxis and bruising since the age of four. Initially, these episodes of recurrent epistaxis were mild and self-limiting. On examination, during more severe episodes, she had multiple large echymoses (variable sizes, most measuring 2x3 cm) present on the trunk and extremities. The patient reported that they occurred both spontaneously and with minimal trauma. After two years of first occurrence, at the age of six, these episodes became more frequent and were accompanied by widespread ecchymoses. Her workup showed a normal platelet count but prolonged bleeding time, an atypical finding for ITP, and she was diagnosed with ITP. 

Over the next two years, her symptoms worsened significantly. At the age of eight, she developed continuous vaginal bleeding for almost eight weeks despite the absence of any secondary sexual characteristics or menarche. During this period, she received daily platelet transfusions. Two months later, she got a splenectomy done for presumed refractory ITP because of recurrent symptoms. Following splenectomy, her symptoms resolved, and she did not require any treatment for almost a year. However, her symptoms returned after this, manifesting as recurrent epistaxis and bruising. During this time, she was started on tranexamic acid and oral corticosteroids; she also received regular platelet and blood transfusions whenever needed. required. She reported improvement in her symptoms after taking these medicines. 

At the presentation with hepatitis C at the age of 17 years, it was suspected that her hepatitis C infection was likely transfusion-related, as she had previously received multiple transfusions (due to her bleeding disorder). Hence, her entire lab workup was repeated to re-evaluate her bleeding disorder. Repeat investigations revealed abnormal platelet morphology, with giant platelets on peripheral blood smears, and the ristocetin test showed no agglutination in its presence. Following this, flow cytometry was done, which showed reduced surface expression of the platelet glycoprotein GPIb/IX/V complex, confirming the suspected diagnosis. Following these lab findings, her case was further discussed amongst the medical team, and she was diagnosed with BSS, which explained her lifelong bleeding tendency and poor response to conventional ITP-directed treatment. 

Table 1 shows a detailed chronological timeline of clinical features, investigations, diagnoses, and management. 

**Table 1:** Chronological timeline of case ITP: immune thrombocytopenia purpura

Age	Clinical Features	Key Investigations	Working Diagnosis	Management
4 years	Recurrent episodes of epistaxis, mild and self-limiting	-	Not established	Observation, symptomatic treatment
6 years	Increased frequency of epistaxis, widespread ecchymoses	Platelet count: Normal range; Bleeding time: Prolonged (more than 20 mins)	ITP	Conservative management
8 years	Continuous vaginal bleeding for 8 weeks	Platelet count: Fluctuating (between 90,000 and 160,000 per microliter); Hemoglobin: Low (7-8 g/dl)	ITP	Daily platelet transfusions
8 years	Ongoing bleeding despite transfusions	-	Refractory ITP	Splenectomy
9 years	Symptom-free period	-	ITP	No active treatment
10-16 years	Recurrent epistaxis, easy bruising	Intermittent thrombocytopenia (dropping to 70,000 per microliter) and anemia (7-8 g/dl and dropping to 6 g/dl once)	Relapsed ITP	Tranexamic acid, oral corticosteroids, platelet and blood transfusions as required
17 years	Persistent bleeding tendency prompts re-evaluation	Giant platelets on peripheral smear, abnormal ristocetin test	Suspected inherited platelet disorder	Advanced platelet function testing
17 years	Platelet adhesion defect confirmed	Flow cytometry: reduced GPIb-IX-V expression	Bernard–Soulier syndrome	Supportive care; platelet transfusion only if symptomatic

Following confirmation of her bleeding disorder, the patient was advised to maintain regular follow-up with Haematology. She is required to have a complete blood count performed monthly, and platelet transfusions are considered whenever her platelet count drops or she develops relevant symptoms. As of date, two years after the diagnosis, she is undergoing regular follow-up. 

## Discussion

As BSS is a rare condition and its clinical presentation is similar to other bleeding disorders, it is often misdiagnosed, especially as ITP [8,9]. Even though ITP and BSS share some overlapping features, BSS should always be considered in patients with refractory ITP. The presence of a prolonged bleeding time, even though the platelet count was normal, provides a strong diagnostic clue against classic ITP and should prompt further investigation towards an alternate diagnosis. BSS can be differentiated from ITP by evaluating platelet agglutination in the presence of ristocetin (defective agglutination in BSS) and by flow cytometry studies [2]. 

This case highlights how individuals with BSS can be misdiagnosed with ITP and undergo procedures like splenectomy [10,11]. Splenectomy in BSS has not shown improvement in bleeding tendency but has improved platelet count [12]. However, it is important to note that this patient reported a transient improvement in symptoms post splenectomy, something that is consistent with previously reported increase in platelet count, likely due to reduced platelet sequestration in the spleen. This effect is short-lived and does not address the underlying qualitative platelet defect, which explains the eventual return of symptoms. Moreover, this case also highlights the potential complications that could be associated with repeated transfusions, such development of hepatitis C, which was likely due to that. This comorbidity could potentially complicate disease management in such patients. However, the current patient was diagnosed and treated for hepatitis C in a timely manner, and her treatment did not pose any reported complications for the underlying bleeding disorder.

Similarly, a case series by Reisi (2020) reports that BSS can be mistaken for ITP [9]. Similar to the current report, Reisi's report also describes patients with early bleeding, easy bruising, and thrombocytopenia who were initially misdiagnosed and treated for ITP with steroids or even splenectomy, but with limited or only temporary improvement. Key features that helped the diagnosis in both the Reisi report and the current one include giant platelets on peripheral smear, prolonged bleeding time, and abnormal ristocetin test results. One important factor that was not looked at in our case was a suggestive family history, especially in the context of parental consanguinity. While Reisi studied several children [9], our case follows a single patient over many years, highlighting the prolonged mismanagement and the importance of advanced platelet testing for definitive diagnosis. Both papers highlight the need to consider BSS in patients with refractory or atypical ITP to ensure appropriate care and avoid unnecessary procedures. 

Monteiro et al. (2021) also illustrate the diagnostic challenges of BSS being misdiagnosed as another bleeding disorder [7]. In their case, however, the patient was misdiagnosed with type 2B von Willebrand disease for three decades. Similar to our case, Monteiro et al.'s patient also had early-onset mucocutaneous bleeding, thrombocytopenia, and giant platelets, and received ineffective treatments over many years [7]. Unlike Monteiro et al.’s patient, whose genetic testing confirmed a homozygous GP1BA variant in late adulthood, our patient benefited from earlier platelet function testing, which guided suspicion toward a congenital platelet disorder. Thus, definitive diagnosis using platelet function testing and genetic analysis (if available) is crucial to avoid unnecessary interventions such as splenectomy or inappropriate factor replacement. 

This case highlights the importance of considering rare congenital platelet disorders like BSS in the differential diagnoses of patients presenting with refractory bleeding and thrombocytopenia. The decade-long journey of this patient with misdiagnosis and unnecessary interventions underscores the need for early utilization of platelet function testing and flow cytometry. This report adds to the existing body of literature on BSS, aiming to enhance awareness and facilitate earlier diagnosis, ultimately improving patient outcomes. Further research and reporting of similar cases can contribute to better diagnostic approaches and treatment guidelines for this rare condition. 

## Conclusions

BSS is a rare inherited bleeding disorder that presents similarly to other bleeding disorders, because of which it can often be misdiagnosed and treated as ITP. This case report highlights the importance of considering alternate and rare platelet disorders in the management of ITP, particularly when clinical red flags such as presentation in early childhood or refractoriness to standard ITP therapies are present. Failure to do this can result in invasive, unnecessary, and non-curative measures like splenectomy. It is essential to consider early diagnosis using platelet aggregation studies with ristocetin and flow cytometry to reduce bleeding and optimize care. 
